# The Differential Weights of Motivational and Task Performance Measures on Medial and Lateral Frontal Neural Activity

**DOI:** 10.1523/JNEUROSCI.0007-22.2023

**Published:** 2023-06-07

**Authors:** Clément Goussi-Denjean, Vincent Fontanier, Frederic M. Stoll, Emmanuel Procyk

**Affiliations:** ^1^Inserm, Stem Cell and Brain Research Institute U1208, Université Lyon 1, Bron, 69500, France; ^2^Nash Family Department of Neuroscience and Friedman Brain Institute, Icahn School of Medicine at Mount Sinai, New York, New York 10029

**Keywords:** cingulate cortex, cognitive control, difficulty, electrophysiology, local field potential, monkeys

## Abstract

Behavioral adaptations are triggered by different constraints given by rules, and are informed by outcomes, or motivational changes. Neural activity in multiple frontal areas is modulated during behavioral adaptations, but the source of these modulations and the nature of the mechanisms involved are unclear. Here we tested how different variables related to changes in task performance and to behavioral adaptation impact the amplitude of event-related local field potentials (LFPs) in the lateral prefrontal and midcingulate cortex of male rhesus macaques. We found that the behavioral task used induced consistently different types of performance modulation: in relation to task difficulty (imposed by the experimental setup), to successes and errors, and to the time spent in the task. Difficulty had a significant effect on monkeys' accuracy and reaction times. Interestingly, there is also a strong interaction between difficulty and trial success on the reaction times variation. However, LFP modulations were mostly related to reaction times, touch position, feedback valence and time-in-session, with little, if any, effect of difficulty. Hence, difficulty modulated performance but not LFP activity. This suggests that, in our experimental design, execution, regulation, and motivation-related factors are the main factors influencing medial and lateral frontal activity.

**SIGNIFICANCE STATEMENT** Adapting decisions might be determined by several mechanisms and might be driven by motivational factors and/or factors inherent to the task at hand. Multiple frontal areas contribute to behavioral adaptations. One current challenge is to understand which information they process contributes to behavioral changes. Diverging views have emerged on whether task demands, like the decision difficulty, or factors linked to incentives to adapt, are driving frontal activity. Here we show that task difficulty had a strong effect on performance (accuracy and reaction times) but little effect on LFP recorded in monkey lateral prefrontal and midcingulate cortex. However, information related to actions, outcome valence, and time-in-session had major influences. Thus, task difficulty modulated performance but not LFP activity in frontal areas.

## Introduction

Animals adapt dynamically to external events and internal stimuli to reactively or proactively improve their own state. The underlying adaptive mechanisms are investigated in experimental conditions by varying, for instance, the multiplicity of options to choose from, their order, the complexity of sensory information relevant to the task, the temporal scale over which information should be integrated for optimal response, or the nature of rewards associated to options (Chen et al., 2008; Quilodran et al., 2008; Kawai et al., 2015; Stoll et al., 2016a). In experimental as well as in natural settings, other factors also influence behavior: intrinsic motivation, satiety, fatigue, boredom, etc. (Stoll et al., 2016b). All those factors can be reflected in performance (i.e., the accuracy or the speed with which task trials are solved) (Procyk and Goldman-Rakic, 2006; Martinez-Garcia et al., 2015).

Multiple facets of behavioral adaptation, through exploration, learning, valuation, and planning, have been theorized or modeled in different ways together with their objective markers, usually by incorporating some interplay between mechanisms devoted to cognitive control and planning on one side, and to performance monitoring on the other (Behrens et al., 2007). Such interplay is an important feature as it forms the core of adaptive regulation and the modulations of accuracy. Another point of interest is the putative anatomo-functional dissociations of performance monitoring and/or motivational processes compared with control or selection mechanisms. The medial frontal cortex, in particular the rostral subdivision of the midcingulate cortex (MCC), is activated during adaptive exploratory behaviors, with activity shown to negatively correlate with fatigue, and seems to be causally involved in integrating information to regulate decision-making (Müller and Apps, 2019; Procyk et al., 2021). Activity in the lateral prefrontal cortex (LPFC) is often correlated with the implementation of behavioral regulation through action or task set selection (Crowe et al., 2013; Donahue and Lee, 2015). Both MCC and LPFC are coactivated during exploratory and adaptive responses (Stoll et al., 2016a). One proposition is that active performance monitoring and evaluative functions performed by the MCC impact LPFC processes, and thus cognitive control, inducing the regulation in control (Shenhav et al., 2013). In this Expected Value of Control theory, signals of multiple sources processed in MCC contribute to an evaluative and selection function that leads to an optimum signal specifying the identity and intensity of control to be implemented (e.g., by the LPFC). One factor proposed to impact MCC activity is task difficulty (Paus et al., 1998; Botvinick et al., 2001; Shenhav et al., 2014; Sarafyazd and Jazayeri, 2019). However, often because of a lack of operational definition of difficulty and to potential confounds, the nature and role of parameters influencing MCC activity have been highly debated (Kolling et al., 2016).

Here we analyzed LFP from MCC and LPFC recordings in a task where difficulty (i.e., a task parameter inducing changes in accuracy and speed) was manipulated. Accuracy was defined in terms of percent of correct responses, and this incidentally correlated with a change in reaction times (RTs). In a past study using the same datasets, we could not find single-unit activity covarying with difficulty (Stoll et al., 2016a). However, because many studies addressing those questions relied on fMRI data, we seek for difficulty related variations in LFP, a signal supposed to be more correlated with BOLD signal variations observed with fMRI (Logothetis et al., 2001). The behavioral task and context also induced different other adaptive phenomena, in reaction to outcomes and depending on the time-in-session. In these analyses, we thus have sought to answer the question: do frontal areas represent difficulty levels in a visual discrimination task, or do they signal other motivational and value parameters? We analyzed the effect of difficulty on monkey's behavior and brain activity. In addition, we tested other motivational parameters that were previously studied (Donahue and Lee, 2015; Stoll et al., 2016a,b) and that could have an influence on frontal activity variation.

LFP amplitude from both MCC and LPFC revealed little, if any, significant correlation with difficulty. In contrast, signals in both frontal areas presented frequent correlation with other behaviorally relevant variables: time-in-session, current and previous feedback valence, RTs, and touch position. We conclude that neural activity in those regions, rather than reflecting difficulty, is involved in monitoring motivational and value signals relevant to behavioral adaptation.

## Materials and Methods

### Subjects and ethics

We report data obtained from 4 male rhesus monkeys (Macaca mulatta). All animals were used for behavioral analyses (A, D, H, and O) and two for electrophysiological recordings (H and A). Monkeys A, D, H, and O were, respectively, 16, 8, 11, and 8 years old at the time of the experiment. Each monkey was paired in a home cage with another monkey. All procedures followed the European Community Council Directive (2010) (Ministère de l'Agriculture et de la Forêt, Commission nationale de l'expérimentation animale) and were approved by the local ethical committee (Comité d'Ethique Lyonnais pour les Neurosciences Expérimentales, C2EA #42). Hydric control protocols were used to train monkeys to perform tasks and to regulate their motivation level, following protocols similar to those described previously (Gray et al., 2016).

### Behavioral context and task difficulty

The behavioral protocol used in this experiment has been published (Stoll et al., 2016a). The protocol combines a so-called work option, a categorization task involving decisions based on visual cues, and a checking option allowing to gather information on a gauge indicating an upcoming bonus reward (Stoll et al., 2016a). The animal could select at will any of these two options at the start of each trial. In the current paper, we focus on the work option, where we could assess specific aspects of behavioral adaptation and task difficulty (see definition below).

During the recording sessions, monkeys were sitting in a primate chair (Crist Instrument) and interacted with a touch screen monitor (Microtouch System). They used their “preferred” hand, as determined from the initial training period, to interact with the screen (Monkeys A, D, and O left-handed; Monkey H right-handed). For Monkeys A, D, and H, visual presentation and touches were recorded using the CORTEX software (NIMH Laboratory of Neuropsychology). For Monkey O, we used Event IDE (EventIDE, www.okazolab.com). Eye position on the screen was recorded using an infrared video system (Iscan).

**Figure 1.**
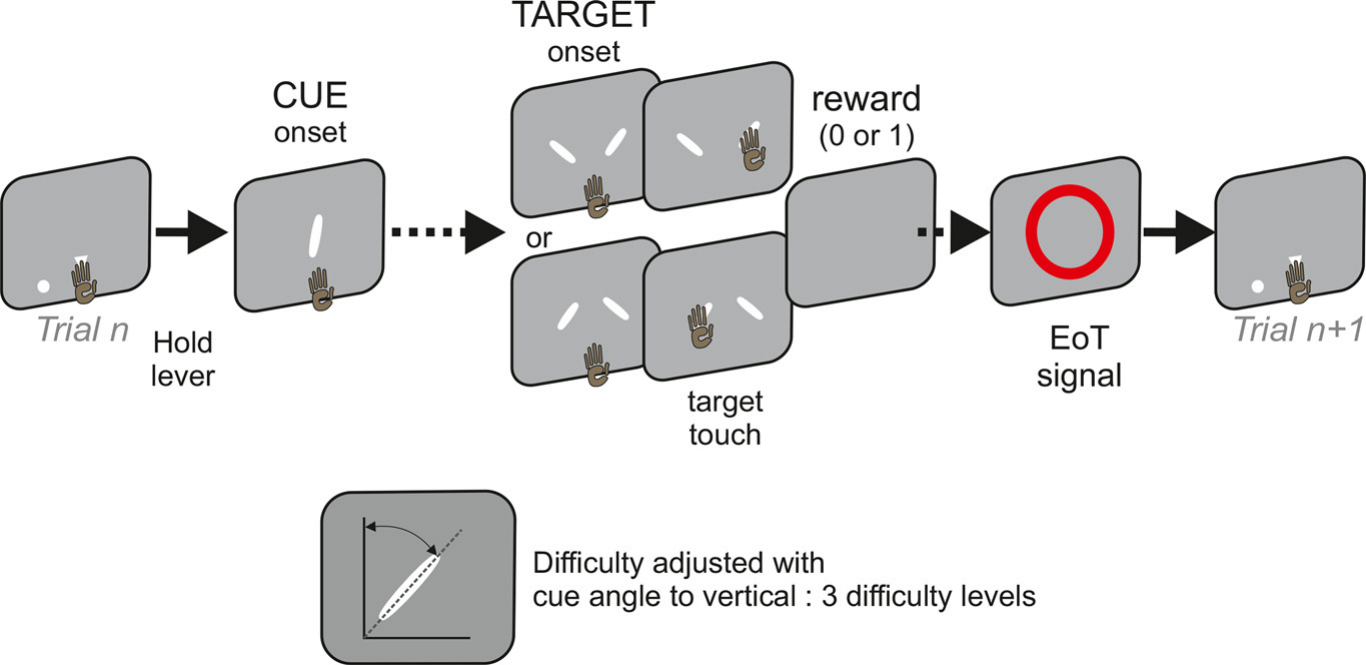
Schematic of the categorization task. A cue (white smoothed bar with upper tip tilted toward the left or the right relative to the vertical) was presented shortly. After a delay, two stimuli (targets) were presented, each being at ±45° from vertical. For 3 of 4 monkeys, the relative position of the rightward and leftward oriented cues was randomly selected (on the left or right of the screen). To be rewarded, the animal had to touch the target oriented in the same direction as the cue. The difficulty of the trial depended on cues' angles. For precisions on timings, see Materials and Methods.

The work option (Fig. 1) was a visual categorization task where a decision to select one of two targets was based on the orientation of a visual stimulus: the animal had to select a target tilted 45 degrees rightward or leftward depending on whether the visual cue was tilted rightward or leftward. The task could be accessed at will by touching and holding a target lever for 700 ms (triangle presented on the lower central part of the screen). During the 700 ms delay, a central white dot was presented, but eye fixation was not necessary. After validating the work lever, monkeys were shown a central smoothed tilted bar (cue display time; Monkey H: 350 ms, Monkey A/D: 600 ms, and Monkey O: 800 ms), followed by a delay (Monkey H: 650 ms, Monkey A/D: 400 ms, and Monkey O: 800 ms). Two targets were then presented, consisting of two oriented bars (45° and −45° from the vertical line) shown on the left and right side of the screen. The animals had 2000 ms to touch one of the targets and were rewarded for touching the one tilted in the same direction as the cue. The left or right placement of the targets, respectively, tilted rightward or leftward was randomized from trial to trial for Monkeys H, O, and D, but not for Monkey A for whom the rightward tilted target was always shown on the right side of the screen. After the touch and a random delay interval of 200-600 ms (step of 200 ms), a central dot was presented for 500 ms and the outcome was delivered (reward or no reward). Cued decisions in the categorization task were rewarded (50% water, 50% apple juice, 0.5 ml; 600 ms) for correct responses or penalized with a timeout (1500 ms) for an incorrect choice; 1500-2000 ms after the outcome onset, a visual stimulus (red circle on for 800 ms, end of trial signal) signaled that a new check versus work choice would be presented after a fixed delay (700 or 1000 ms for Monkey A and H/D/O, respectively). If the animal did not start the trial (no start) for 10 s, or broke timings by releasing the lever too early (break), then the trial was aborted and another trial was initiated.

The difficulty of categorization trials varied depending on three possible absolute angles of orientation (relative to the vertical axis) of the cue, which were pseudo-randomly selected for each trial. Cue stimuli orientations were not fixed across sessions but were defined on each session based on the animal's accuracy in the 5-10 preceding sessions. Two cue orientations (leftward and rightward) for each difficulty level (one combination example for each Monkey D: ±20°, 15°, 10° for easy, medium, and hard trials, respectively, Monkey H: ±20°, 10°, 5°, Monkey A: ±40°, 25°, 10°; Monkey O: ±35°, 25°, 15°) were picked to get, respectively, 90%, 80%, and 70% of correct responses on average. Hard refers to trials using cue stimuli with a small angle relative to the vertical and inducing the lowest level of accuracy. These angle adaptations were used to preserve the same level of uncertainty during this task (in terms of probability of correct response) and counteract potential changes in accuracy across sessions.

### Surgery and signal acquisition

Monkeys A and H were implanted with a head-restraining device in a first surgery. Next, in a second surgery, a craniotomy was performed to expose an aperture over the PFC where a recording chamber was implanted (Gray Matter Research). Analgesics and antibiotics were administered before, throughout, and after surgery. The chamber was kept sterile with regular cleaning and sealed with sterile caps.

Electrophysiological data were recorded using an Alpha-Omega multichannel system (AlphaOmega Engineering). Recording chambers were centered at anteroposterior coordinates 34.4 and 33.6 relative to ear bars (for Monkeys A and H, respectively). Electrophysiological activity was recorded using epoxy-coated tungsten single electrodes (1-2 MO at 1 kHz; FHC) independently lowered using Microdrive guidance (AlphaOmega Engineering). Neuronal activity was sampled at 22 kHz resolution. LFPs were obtained using the signal filtered from 0.5 to 250 Hz (see below). Recordings were referenced on the guide tubes in contact with the dura. Two to six electrodes were used simultaneously to record neuronal activity in MCC and LPFC. Recording sites were reached through a 1 mm spaced recording grid (for details, see Stoll et al., 2016a).

Recording sites in MCC were in the dorsal bank and fundus of the cingulate sulcus at rostro-caudal levels equal or anterior to the level of the genu of the arcuate sulcus. Recording sites in LPFC were located between the posterior end of the principal sulcus and around the arcuate sulcus (areas 6DR, 8B, 8A, and 9/46) and at <3 mm from cortical surface. Frontal areas were targeted preoperatively using Brainsight neuronavigation (Rogue Research) using each animal's anatomic MRI (T1, 1.5 T and voxel 0.6 mm iso). Recording chambers were implanted contralateral to the arm used using online targeting with neuronavigation. Reconstructions of cortical surface, of MRI sections perpendicular to recording grids, and of microelectrode tracks were performed using the same reference frames. Maps and recording locations in the 2 monkeys were combined by aligning grids on the level of the genu of the arcuate sulcus, taken from coronal sections on the MRI and then relocated using views perpendicular to the recording chamber axis.

### Analysis of task performance

All analyses were performed using R (version 3.6.3) with the RStudio environment (R core team, 2014; RStudio Team, 2016).

We analyzed all sessions in which the average accuracy in the categorization task was ≥60%, which left 28, 63, 81, and 101 sessions, respectively, for Monkeys D, H, O, and A. Using the statistical models detailed below, we assessed the following fixed effects that might describe behavioral adaptations on a trial-by-trial basis:

- Difficulty [1, 2, 3]: continuous variable representing the level of difficulty of a trial (easy, medium, or hard) corresponding to one of the three cue angles.
- Time-in-session: the time of the trial in the session normalized between 0 and 1 (Eq. 1 below).
- RT: the time duration between target onset and lever release measured in the trial. RT values were centered (mean RT across sessions for each animal was subtracted) and log-transformed.
- Touch position [left, right]: the target position selected by the animal in the trial.
- Previous feedback [positive, negative]: the feedback obtained by the animal in the previous trial.
- Previous trial [correct, incorrect, no start, break]: nature of the previous trial.
- Accuracy [correct, incorrect]: the performance of the animal in the current trial. Dependent variable used to estimate effects influencing performance.
- Session: day of acquisition of data.

As in our single unit study (Stoll et al., 2016a), we selected multiple factors to assess LFP variations during performance of the categorization task. We included factors only on the basis that they were relevant to the time window of interest (e.g., feedback valence after feedback delivery to assess feedback encoding, but not before).

### Behavioral accuracy variation

During the experiment, difficulty levels were regularly adjusted according to monkeys' accuracy. This putatively allowed to maintain the same amount of cognitive demand all along the experiment. Accuracy is here defined by the ability to correctly categorize the orientation of the cue. We used mixed-effects binomial generalized linear models (glmm, *glmer* in R) on repeated measures to assess the effect of difficulty and of other major factors, such as time-in-session, on accuracy. Because the difficulty level was experimentally controlled based on accuracy, testing the difficulty effect on accuracy served as a data sanity check.

Where indicated, we used data transformation to normalize values between 0 and 1 using the following formula:

(1) $$x(\mathrm{norm})=\frac{x-\text{min}(x)}{max(x)-\text{ min}(x)}$$

The statistical models assessing accuracy variation were logistic regressions of the following form:

(2) $$\begin{array}{l} \text{Logit}\left(\text{E}\left[\text{Accuracy}\right]\right)=(\beta_{0}+{\text{ b}}_{0,\text{S})}+\beta_{\text{1}}\text{Difficulty }+\beta_{\text{2}}\text{Time}-\text{in}-\text{session }+\beta_{\text{3}}\text{Previous feedback }+\\ \left(\beta_{\text{Ts}}+{\text{ b}}_{\text{1},\text{S}}\right)\text{Time}-\text{in}-\text{session }+\varepsilon \left(\text{t}\right) \end{array}$$

where Logit(p) = log_e_(p/1 – p)

and E(Accuracy) is the expected value of Accuracy and Accuracy is the vector of performance in trials (0 if incorrect, 1 if correct), β_0_ and b_0_ are the intercepts for, respectively, the fixed and the random factors, Difficulty is the level of the current trial, Previous feedback, Time-in-session, and ε corresponding to the residuals.

We also included session (S) and time-in-session as random factors. Random factors allowed the model to fit a linear trend for each session accounting for potential intersession variability in time-in-session effect. Models were tested on each monkey individually.

### RT modulations

RTs, which corresponds to the delay between the appearance of the two targets and the release of the lever, were measured, centered to the mean and log-transformed. To assess RT variation during the categorization task, we used a linear mixed model (*lmer*) on repeated measures, with session and time-in-session as random factors:

(3) $$\begin{array}{l} \text{RT }=\left(\beta_{0}+{\text{ b}}_{0,\text{S}}\right)+\beta_{\text{1}}\text{Accuracy x }\beta_{\text{2}}\text{Difficulty }+\beta_{\text{3}}\text{Time}-\text{in}-\text{session }+\beta_{\text{4}}\text{Previous trial }+\\ \left(\beta_{\text{Ts}}+{\text{ b}}_{\text{1},\text{S}}\right)\text{Time}-\text{in}-\text{session}+\varepsilon \end{array}$$

where Accuracy is the nature of the monkey choice (0 if incorrect, 1 if correct), β_0_ and b_0_ are the intercepts for, respectively, the fixed and the random effects. Difficulty, previous trial, time-in-session, are fixed effects, and ε(t) corresponds to the residuals. Subscripts are coded as follows: Ts, time-in-session; S, session. The random-effects capture variation across the multiple sessions and the influence of on the intercept and the slope of the RT change as a function of time-in-session.

The interaction effect of difficulty (three levels) and accuracy on the RT variation was tested in this analysis. We also tested the effects of time-in-session and nature of the previous trial (correct, incorrect, break, and no start) on the RT variations.

To verify statistically whether time periods impacted difficulty and accuracy effects, we divided trials of each session into terciles (beginning, middle, end) with equal number of trials, and applied *glmm* (Eq. 3).

### Relationship between RTs, and accuracy

Analysis of RT and accuracy (percent of correct response) can be performed both across behavioral sessions and session by session. To assess behavioral variability, we tested the relationship between RT and accuracy within each session. A χ^2^ homogeneity test was used to assess the relationship distribution of RT and accuracy for each monkey.

### Analyses of LFP variations

#### Signal preprocessing

We analyzed variations of LFP in MCC and LPFC around cue onset, lever release, and feedback. Based on the behavioral results showing trial-to-trial regulation and within-session regulation of accuracy and RT, we tested whether those effects and difficulty correlated with LFP measures. Our dataset contained 288 LFP recording sites in MCC (Monkey H: 106; Monkey A: 182) and 173 in LPFC (Monkey H: 66; Monkey A: 107).

Artifacts corresponding to large disruptions of the signal were detected on the segmented raw data and removed manually from epochs of interest using the MATLAB FieldTrip toolbox (Oostenveld et al., 2011). The raw signal was bandpass-filtered between 0 and 100 Hz, and the line noise was removed by applying a bandstop filter at 50 and 100 Hz frequencies using the FieldTrip toolbox. We extracted the signal recorded within the 2000 ms around the three events of interest. We downsampled the data at 100 Hz for event-related analyses.

Three time epochs were analyzed: the 2000 ms following the cue onset (appearance of the tilted bar on screen), the 1000 ms before and after the lever release, and 2000 ms following the feedback delivery (juice or nothing).

Depending on the focus of the analyses (assessing within-trial effects or between-trial effects), we applied or not a pre-event baseline. This consisted in removing for each trial, amplitude values of the LFP before the onset (0-100 ms) of the given event (baseline) to the LFP values on the time window of interest. For instance, no baselining was used to test previous feedback or time-in-session (across trials) effects that are potentially evolving between trials. Inversely, a within-trial baseline was applied to assess touch position, difficulty, or RT. For cue onset and lever release, the baseline time window encompassed 0-100 ms before the cue onset; for the feedback, 0-100 ms before the feedback delivery.

#### Statistics

To assess which factors significantly influenced the amplitude of the LFP, we used a linear regression model coupled with a Type II ANOVA. For cue onset and lever release events, we regressed five factors in linear models: RT, difficulty, time-in-session, previous feedback, and touch position to assess their effect on the LFP signal variation in individual sessions.

We used the simplest additive model without interaction to test factors' effects:

(4) $$\begin{array}{l} \text{LFP}\left(\text{t}\right)=\beta_{0}\left(\text{t}\right)+\beta_{\text{1}}\left(\text{t}\right)\text{RT }+\beta_{\text{2}}\left(\text{t}\right)\text{Difficulty }+\beta_{\text{3}}\left(\text{t}\right)\text{Time}-\text{in}-\text{session }+\beta_{\text{4}}\left(\text{t}\right)\text{Previous feedback }+\\ \beta_{\text{5}}\left(\text{t}\right)\text{ Touch Position }+\varepsilon \left(\text{t}\right) \end{array}$$

For the feedback event, we included a “feedback” fixed effect (positive or negative) to the other five factors. We assessed fixed effects on individual sessions because recording positions were different across sessions. We also assessed the effect of the previous feedback valence on the LFPs in the current trial.

To estimate the likelihood of finding significant effects by chance, we ran the different linear models on permuted data. Permutations were done by running the same models, for each time window of interest, 100 times with shuffled factors levels. The permutation data were used to compute the probability of finding each proportion of significant sites per time bin for each fixed effect. The proportion found was finally thresholded at *p* < 0.01.

In particular, for time-in-session statistics, we investigated whether effects were mostly going in one direction (e.g., LFP amplitude mostly increasing with time-in-session). To evaluate such biases, we extracted the distribution of *z* values from *glm* analyses for each time bin for a particular fixed effect and computed the skewness of the distribution of *z* values. Skewness is a measure of symmetry and is based on the second and third central moments of a distribution. Positive values of skewness indicate a distribution skewed on the right. As a rule of thumb, absolute values of skewness >1 indicate highly skewed data, between 0.5 and 1 moderately skewed, and <0.5 approximately symmetric.

We wanted to assess whether the relationship between RT and LFP was stable over time. To answer this, we split data from each session in two equivalent periods that contained the same number of correct trials (beginning and end periods). For each of these periods, we separated trials into quartiles of RT and calculated, 1 s around the release, the mean LFP for each period and each RT group, and depending on the sign (positive or negative) of the correlation between RT and normalized LFP (Eq. 1). Finally, we subtracted, session by session, the *z* value of the second and first periods to index the change in the RT to LFP relationship over time. We tested changes with one sample *t* test difference from mean(µ) = 0.

Aside from linear model analyses, we also tested all fixed effects with nonparametric Kruskal–Wallis tests. Results were similar and hence not reported in the paper.

### Time frequency analyses

Finally, we performed time-frequency analyses on signals recorded between the cue onset and the time of lever release. The *ft_freq_analysis* function from the FieldTrip toolbox was used to perform a wavelet time-frequency transformation (convolution with complex Gaussian Morlet's wavelets with a ratio f/δf of 7). This analysis was done on the range 2-100 Hz in 2 Hz steps and in 100 ms windows. Statistical models identical to those used for LFP amplitude were applied to test whether oscillatory power in various frequency bands varied with fixed effects (difficulty, touch position, RT). As for LFP amplitude, we assessed the frequency of factors effects across all sites. We then computed the average percent of sites with significant factor effects within four 500 ms time windows after cue onset (0-2 s) and around lever release (−1 to 1 s) for the 2 monkeys and the two brain regions (LPFC and MCC). We measured the average percentage of sites for which the power in the theta (4-8 Hz) and beta (12-30 Hz) bands was significantly modulated for each factor.

## Results

### Regulations of performance

First, we assessed the factors influencing accuracy in the task, whether related to specific properties of the task (e.g., difficulty to categorize the visual stimulus) or inherent to the adaptation of behavior, after outcomes (e.g., post-error adaptation) and/or within-sessions following motivational changes.

**Figure 2.**
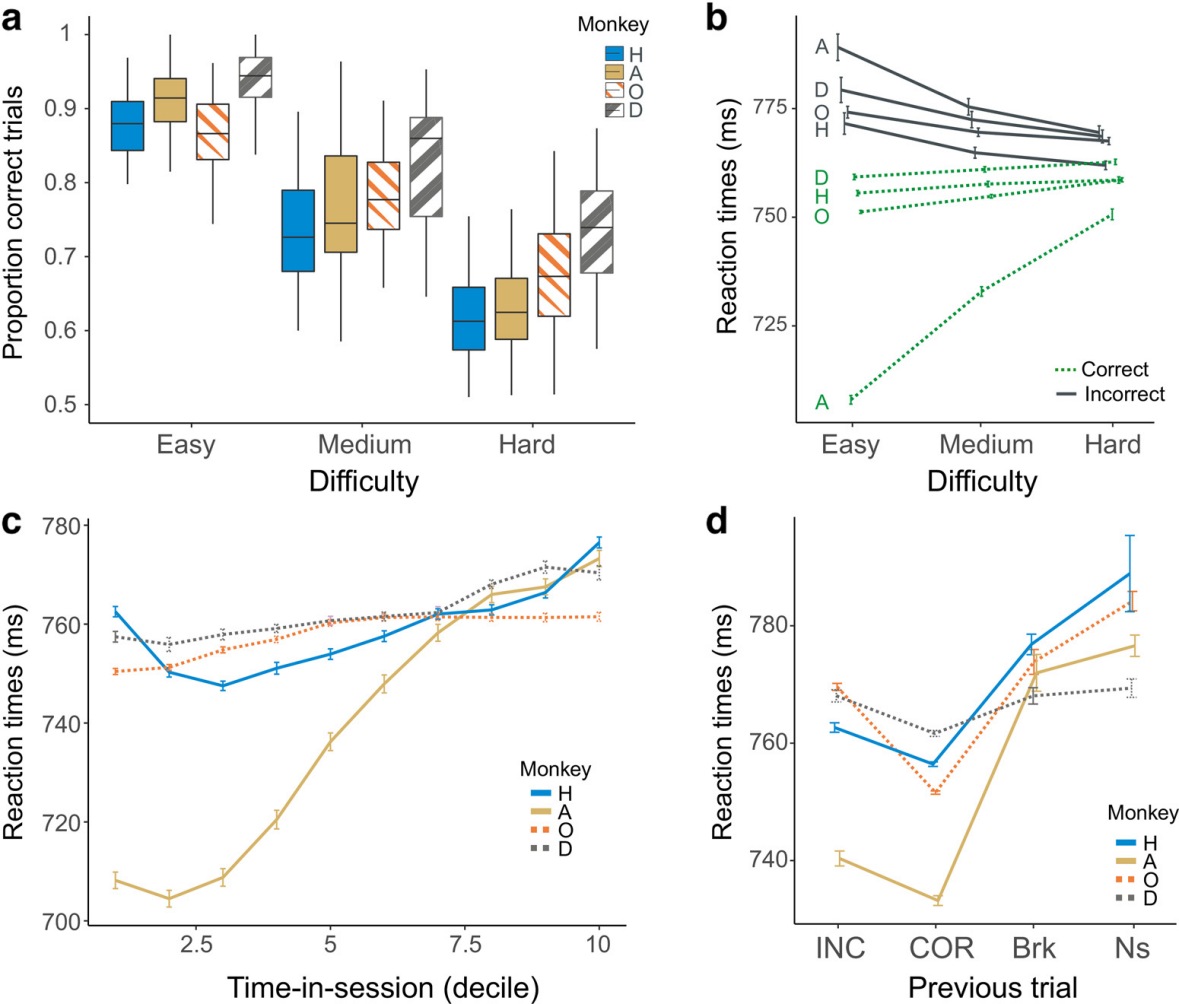
Accuracy and behavioral adjustments: data from 4 monkeys. ***a***, Proportion of correct responses depending on difficulty levels (3 absolute angles of the cue). Difficulty has been controlled based on accuracy for each animal independently. ***b***, Average of normalized RT (+/− SEM) in correct (green, dashed lines) and incorrect trials (gray, plain lines) for the different difficulty trials. Note the opposite relationship (interaction) between RT and difficulty for correct and incorrect trials. ***c***, Time-in-session effect observed in RT changes across sessions. Average RT (+/− SEM) were measured for deciles of each session duration and averaged across sessions. ***d***, Adaptive reaction to previous trial types. The figure presents average RT (+/− SEM) for responses in trials following incorrect nonrewarded (INC), correct rewarded (COR), nonrewarded break in timing (Brk), and nonengaged trials (Ns). Note, in particular, the post-error slowing (compared with post-correct response).

**Figure 3.**
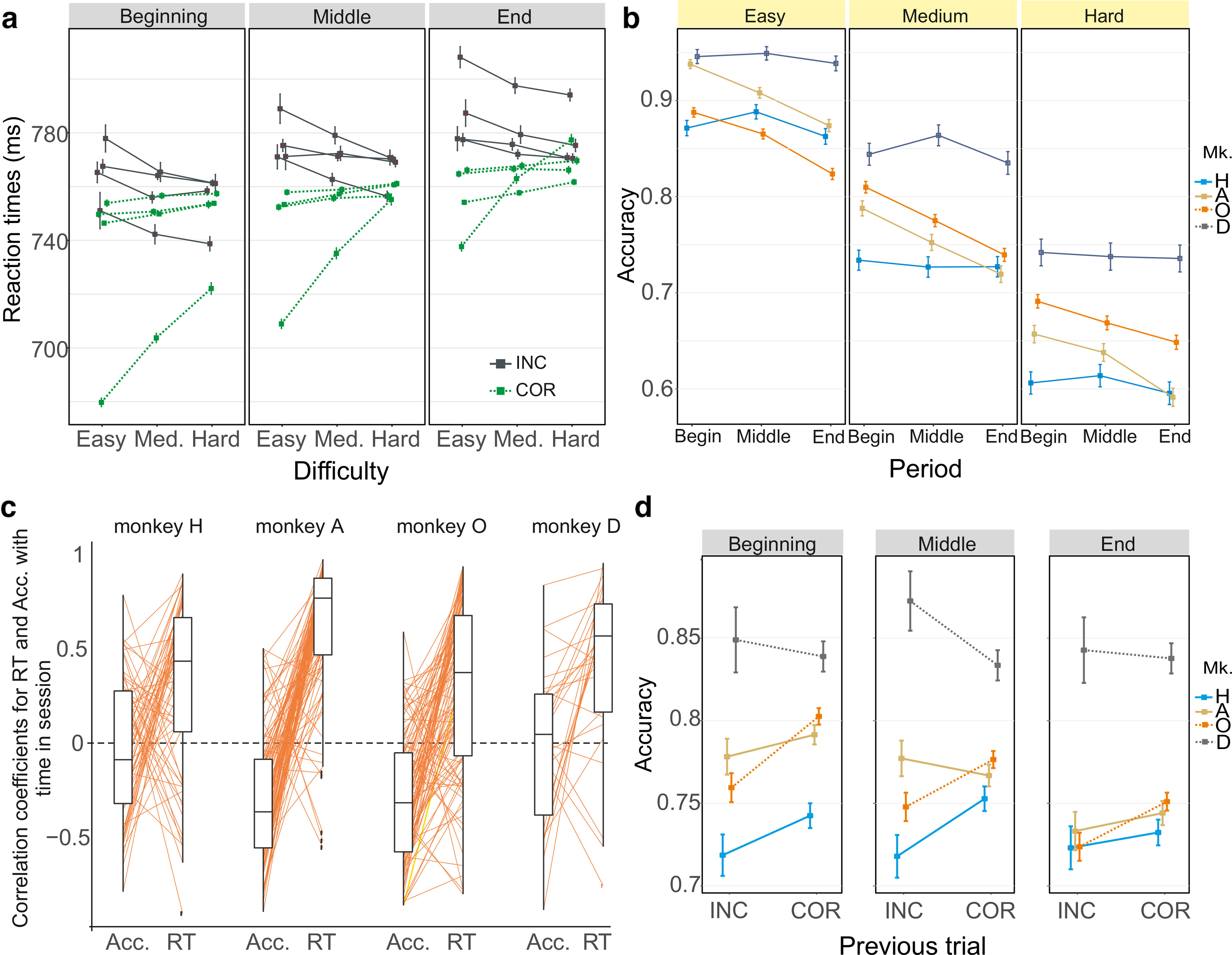
Performance within sessions. ***a***, Average RT (+/− SEM) in correct (green, dashed lines) and incorrect trials (gray, plain lines) for the different difficulty trials and for three successive periods covering sessions (beginning, middle, and end periods) for each monkey. The opposite relationship between RT and difficulty for correct and incorrect trials was stable across the three periods for all monkeys. ***b***, Accuracy for each difficulty level averaged for the three time periods of sessions. ***c***, Relationships between within-session changes in RT and within-session changes in accuracy (Acc.) for each monkey. Box plots represent the global populations of correlation coefficients for RT versus time-in-session and Acc. versus time-in-session. Each thin line links the values associated with each session. ***d***, Average accuracy for each monkey depending on the previous feedback at the beginning, middle, and end stages of sessions.

Our task included three levels of difficulty, designed experimentally (see Materials and Methods) to elicit different levels of performance. Effectively, accuracy (proportion of correct responses) decreased with increased level of difficulty, that is, for stimuli bars oriented closer to the vertical (∼90°) (Fig. 2*a*; Eq. 2, logistic regression; *p* < 0.05 for all monkeys; *z* = −30.8, −40.7, −35.6, and −21.0 for Monkeys H, A, O, and D, respectively), confirming the efficiency of the procedure. More importantly, RT not only varied with difficulty but were also influenced by the accuracy of the trial. This was revealed by a significant interaction between difficulty and accuracy (correct or incorrect) on RT (Fig. 2*b*; Eq. 3; *p* < 0.05 for all monkeys; *z* = −5.9, 16.7, 8.7, and 5.7 for Monkeys H, A, O, and D, respectively). Average RT increased with difficulty when monkeys performed correctly, while average RT decreased with difficulty when monkeys failed the categorization. This pattern was very stable and observed throughout sessions (Fig. 3*a*; Eq. 3; significant interaction between difficulty and accuracy for the three time periods for all monkeys, *p* < 0.05). In correct trials, the positive correlation between RT and difficulty was expected and might be explained by increased cognitive demand and engagement of control for difficult angles, leading to increased time spent in computing and resolving the uncertainty associated with the cue. The inverse effect for incorrect trials was unexpected but could relate to a different phenomenon. Incorrect responses were more numerous for hard than easy trials, and errors in those trials might have different origins. One possibility is that the majority of errors in easy trials were because of lapses of attention and/or motivation, hence leading to increased time to react and respond.

Interestingly, all 4 monkeys showed a time-in-session effect characterized by a within-session increase in average RT (Fig. 2*c*; Eq. 3; *p* < 0.05 for all monkeys; *z* = 5.7, 15.2, 5.7, and 6.1 for Monkeys H, A, O, and D, respectively). The accuracy remained stable for 2 monkeys (H and D) and decreased (Acc^–^) for the other two (Fig. 3*b*; Eq. 2; *p* < 0.05 for Monkeys A and O, respectively; *z* = −8.7 and −9.8, respectively). We defined a session to be RT^+^ versus RT^–^ and Acc^+^ versus Acc^–^ depending on the positive or negative relationships between RT, or accuracy, with time-in-session. The positive correlation between RT and time (RT^+^) was, in many cases, associated with a decreasing accuracy within the session (in 77%, 56%, 49%, and 35% of sessions for Monkeys A, O, H, and D, respectively) (Fig. 3*c*). The proportion of sessions with such a trend (RT^+^/Acc^–^) was significantly different from the proportion of sessions with the three other possibilities (RT^–^/Acc^+^; RT^+^/Acc^+^; RT^–^/Acc^–^) for Monkeys A and O (χ^2^ homogeneity test; *p* < 0.05; χ^2^ = 23.9 and 12.9 for Monkeys A and O, respectively, and not significant, χ^2^ = 5.6 and 4.6 for Monkeys H and D, respectively). Such time-in-session effects have been previously observed in humans and animals and correlate in some conditions with changes in frontal neural signals, suggesting relationships with variations in motivational or cognitive control (e.g., Stoll et al., 2016b).

Post-error adaptation was also observed for 3 monkeys. RTs were higher in trials following erroneous responses compared with correct ones (Fig. 2*d*; Eq. 3; *p* < 0.05 for Monkeys H, O, and D; *p* = 0.414 for Monkey A; *z* = −7.6, −0.8, −30.9, and −3.8 for Monkeys H, A, O, and D, respectively). Such slowing of RT was also observed after execution errors (Fig. 2*d*; Eq. 3; trials in which monkeys responded too late or too early, and were thus nonrewarded [Brk]; *p* < 0.05 for all monkeys; *z* = 9.3, 2.9, 2.6, and 2.4 for Monkeys H, A, O, and D, respectively) compared with post-correct response. Such effects have been described in past studies, including in monkeys, and have been correlated with changes in frontal neural activity (e.g., Quilodran et al., 2008). Accuracy was also affected by the previous trial correctness, where monkeys were more likely to be correct following a correct compared with incorrect trials (Fig. 3*d*; Eq. 2; *p* < 0.05 for Monkeys O and D, *p* = 0.07 for Monkey H, *p* = 0.873 for Monkey A, *z* = 1.81, −0.16, 3.31, and −2.82 for Monkeys H, A, O, and D, respectively).

### Frontal LFP are modulated by behavioral adaptation and motivation, not by difficulty

Models of cognitive control regulation suggest that multiple incentives or driving variables are monitored and integrated to derive a value of exerting control (Shenhav et al., 2013). Such variables (e.g., expected value of outcomes, motivational incentive, uncertainty, or difficulty of the task) might be integrated in MCC and used as a modulatory factor in LPFC, but evidence for such integrations is scarce.

**Figure 4.**
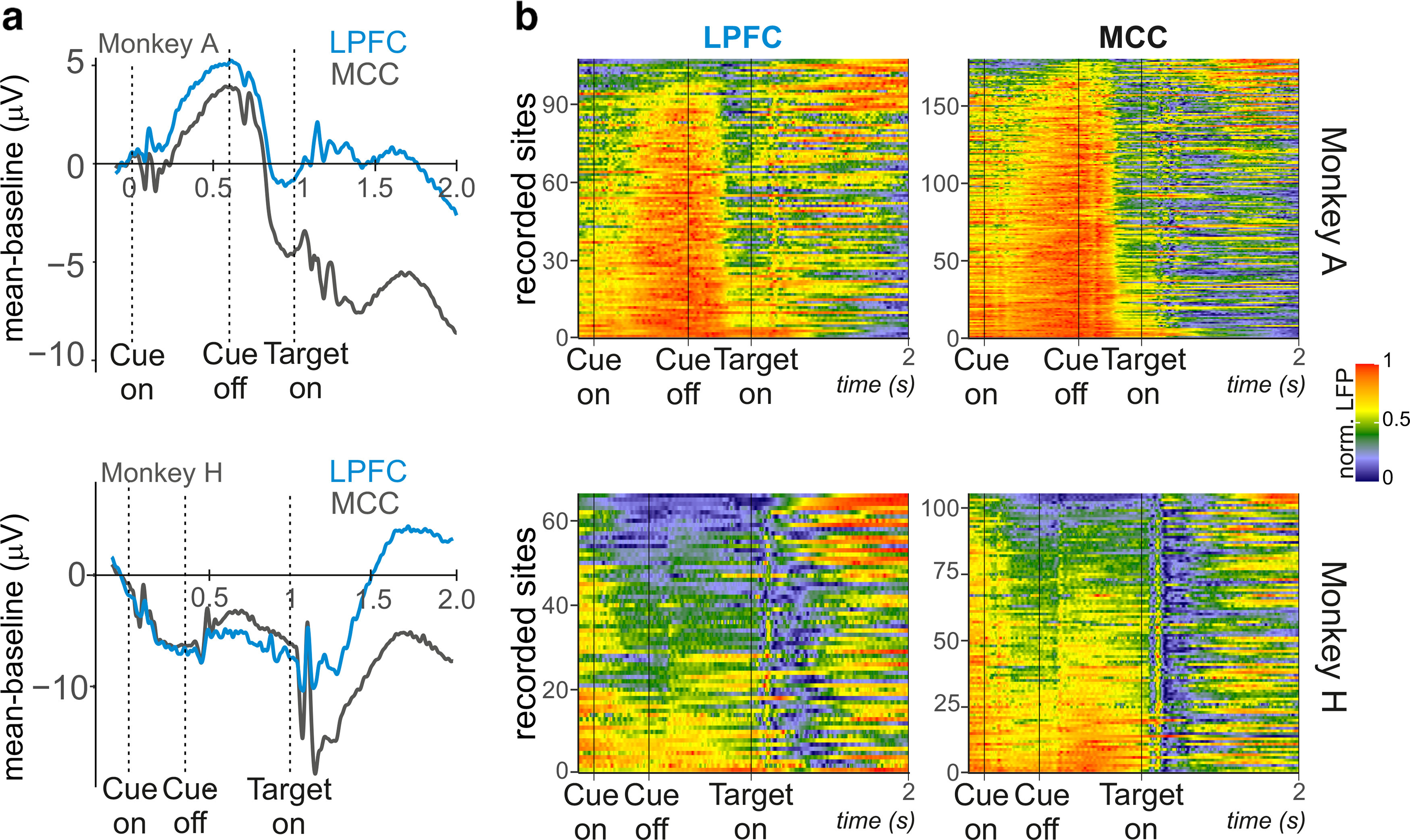
Normalized LFP signal presented from cue to target onset. ***a***, All recordings from MCC (black) and LPFC (blue) were baselined and averaged. Each panel corresponds to data from Monkey A (left) and Monkey H (right). The LFP signal was baselined to the 100 ms preceding the cue onset. ***b***, Averaged normalized LFP represented for all recording sites (*y* axis) for each area (left, MCC; right, LPFC) and monkey (top, Monkey A; bottom, Monkey H).

We assessed LFP variations in MCC and LPFC around three task periods, respectively, associated with information collection, decision, and feedback monitoring. Averaged of all LFP traces, for each monkey and each area aligned on the cue onset, are represented in Figure 4*a*. Individual session details are represented alongside showing intersession variability (Fig. 4*b*). Signals dynamics show a reproductive pattern across recordings, sessions, and between frontal areas.

**Figure 5.**
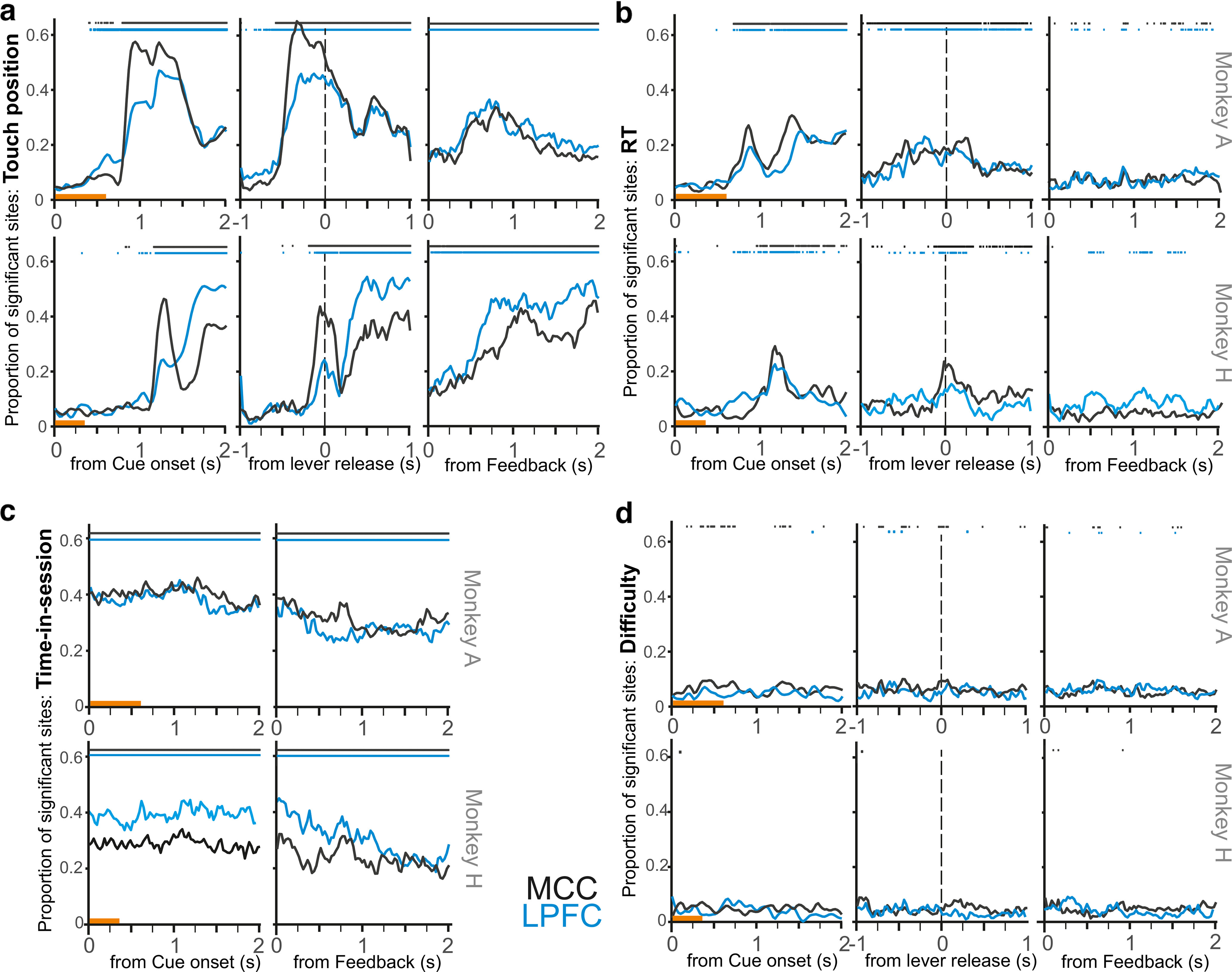
Fixed effects on LFP modulation after cue onset, around lever release, and after feedback onset. Each plot represents the proportion of sites that presented a significant modulation for the respective fixed effects, that is, touch position in ***a***, RT in ***b***, time-in-session in ***c***, and difficulty in ***d***, and at each time bin. Data are shown for MCC (black) and LPFC (blue). ***a***, ***b***, ***d***, The signal was referenced to baseline (pre-cue). ***c***, Time-in-session fixed effect across trials was estimated on nonreferenced signals. Significance at *p* < 0.01 against permutations is shown on top of each graph. Orange line above the abscissa indicates the display of the cue. ***d***, Difficulty shows an effect on <10% of sessions in MCC and for very limited time periods.

We first measured the influence of both within-trial (difficulty, touch position, RT) and between-trial variables (previous feedback [post-error reaction] and time-in-session) on LFP. We report the main outcomes (Eq. 4, proportion of significant sessions with *p* < 0.05) from time-resolved analyses using linear models containing the relevant fixed effects: touch position, previous feedback, difficulty, time-in-session, and RT (Fig. 5).

### Modulations with touch position and RT

The amplitude of LFP was significantly modulated by the position of the chosen target in more than half of MCC and LPFC recordings (Fig. 5*a*). The latency of the rise in the number of significantly modulated sites reflected the respective versions of the task performed by the 2 monkeys. Indeed, the effect of touch position started earlier for Monkey A (after 750 ms for the MCC and the LPFC) who could anticipate response locations as leftward and rightward targets were always in the same position (see Materials and Methods), but later for Monkey H who had to wait for the target onset (after 1000 ms). Interestingly, touch position was an influential factor after feedback onset in both MCC and LPFC.

MCC and LPFC LFP variations were also influenced by RT around lever release. As for touch position, the main effect appeared earlier for Monkey A than Monkey H (Fig. 5*b*).

### Modulations with time-in-session

For both monkeys, time-in-session had a sustained effect on LFP amplitude as observed for the two epochs of interest (after cue onset and around lever release) (Fig. 5*c*). This was one of the main factors of influence, being significant in 30%-40% of sessions in both areas. We observed a systematic bias for positive effects of time-in-session (i.e., increased LFP amplitude with time-in-session), especially strong for MCC recordings (skewness of *z* values distributions, 1 value per bin, for post-feedback intervals: 1.20 and 1.78 for MCC, and 0.55 and 0.61 for LPFC, in Monkeys A and H, respectively). Because RTs also evolved over time and RTs were influencing LFP amplitude, one could wonder whether RT effects were confounding time-in-session effects. First, the statistics for RTs were weaker than for time-in-session and did not reveal any bias along sessions (skewness of *z* values distributions for post-feedback intervals: −0.05 and 0.18 for MCC, and 0.17 and 0.10 for LPFC, in Monkeys A and H, respectively). Second, effects for RT and time-in-session were negatively correlated, with RT effects slightly increasing when time-in-session effects were lower (Spearman correlation on *z* values for post-feedback intervals, *p* < 0.001, ρ = −0.11 (*n* = 36,582) and −0.28 (*n* = 21,306) for MCC, and −0.08 (*n* = 21,507) and −0.17 (*n* = 13,266) for LPFC, in Monkeys A and H, respectively). Together, this suggests that the influence of time-in-session and RT on LFP amplitudes were not confounded.

**Figure 6.**
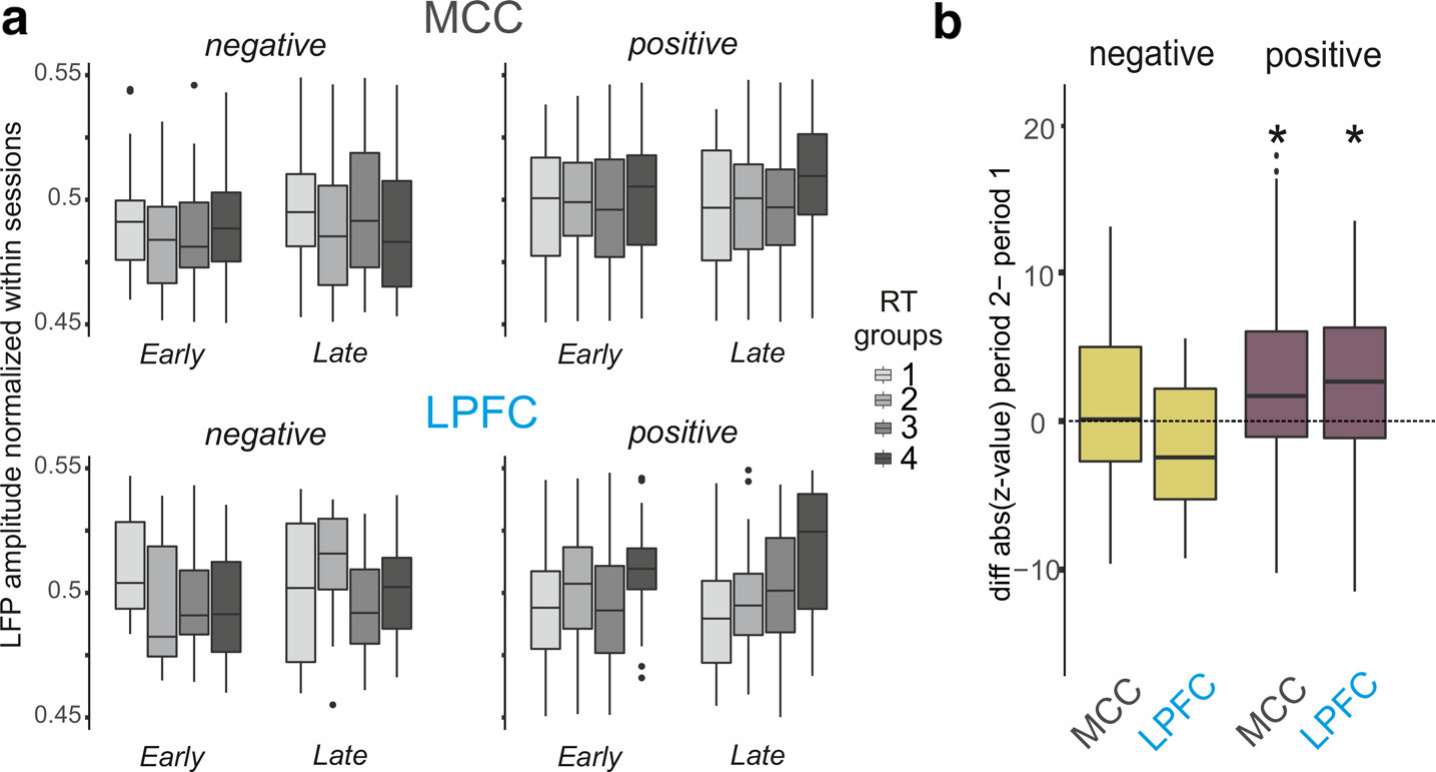
LFP variation depending on RT variation and time period. ***a***, Boxplots of LFP averaged value for 4 RT range values (quartiles) and for two periods of time (early and late in session). Data were split depending on the relationship between RT and LFP. Left, The negative β values group. Right, The positive β values group. ***b***, *z* value differences between the two time periods. A positive value indicates that the influence of RTs on the LFP was stronger early in the session than late in the session. The difference is higher when the LFP amplitude increases with RT value (positive relationship).

LFP modulation with time-in-session could relate to compensatory mechanisms taking place along sessions to maintain performance in the task. In such a case, one could expect correct trials with similar RT toward the end of sessions to be associated with stronger fluctuations in LFP than their counterparts at the beginning of sessions. We looked for such an effect and found that the relationships between RT and LFP changed over time (Fig. 6). A majority of sites showed a positive correlation between RT and LFP amplitude, while only a few showed a negative correlation (for LPFC: 78% and 70% of positive sites, respectively, for Monkeys A and H; for MCC: 72% and 55% of positive sites, respectively, for Monkeys A and H). Positive correlations between RT and LFP amplitude increased in late compared with early part of the session (see Materials and Methods) for both MCC and LPFC. No difference was observed for negative correlations (Fig. 6; one-sample *t* test; *p* < 0.05 for positive *z* values and *p* > 0.05 for negatives ones).

### LFP changes in relation to outcomes

**Figure 7.**
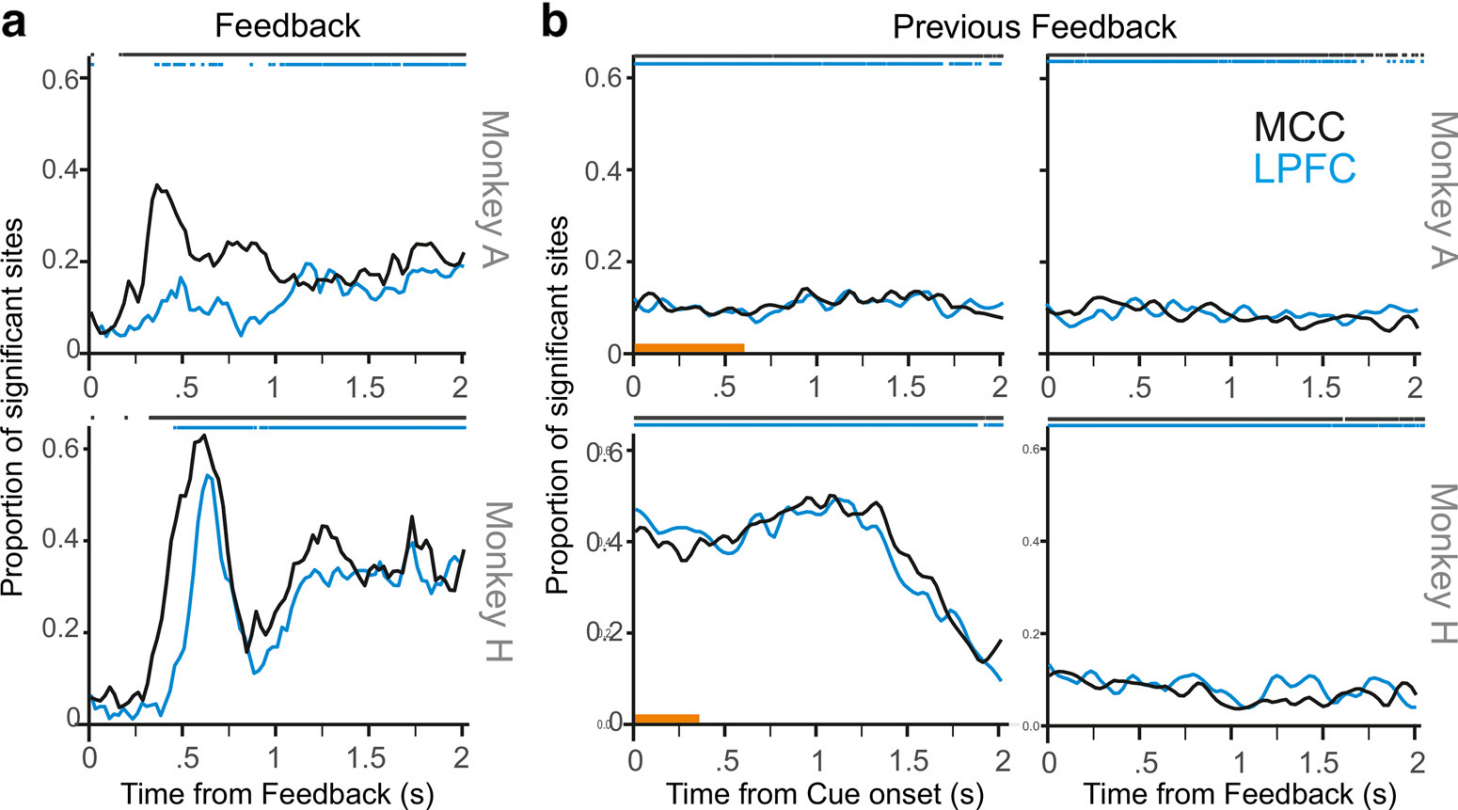
Feedback valence and LFP modulations. Graphs represent the proportion of sites with a significant effect of feedback valence (positive/reward or negative/no reward). ***a***, Analyses performed on signals referenced to pre-feedback time and aligned at feedback onset. ***b***, Effect of previous feedback (positive or negative) on the current trial LFP amplitude aligned on cue onset (left) and next feedback (right) (nonreferenced signal). Significance at *p* < 0.01 against permutations are shown on top of each graph. Orange line above the abscissa indicates the display of the cue. Feedback valence is encoded from ∼200 ms after onset with a peak of influence (in ***a***), followed by a sustained encoding observable in the next trial (in ***b***).

As expected, when aligned on feedback onset (time of reward or no reward), the feedback valence had a major influence on LFP amplitude, reflecting the onset (Fig. 7*a*) of feedback-related potentials and during the intertrial period (Fig. 7*b*). The effect of feedback valence also appeared earlier in MCC than in LPFC (Fig. 7*a*). This is in line with several past studies showing precedence of MCC over LPFC feedback-related activity in single-unit activity or high γ oscillations (Rothe et al., 2011; Stoll et al., 2016a).

The influence of the outcome was observed throughout the intertrial interval (Fig. 7*a*) and in the following trial until the next response (at ∼1.5 s after cue; Fig. 7*b*), although with higher impact for 1 of the 2 animals. This could be tentatively associated with the differential effect of the previous feedback on RT (Fig. 2*d*; Eq. 3; *p* < 0.05 for Monkey H; *p* = 0.414 for Monkey A) and on accuracy in these 2 animals (Fig. 3*c*; Eq. 2; *p* = 0.07 for Monkey H, *p* = 0.873 for Monkey A). Such sustained effect of feedback valence indicates that a trace from the previous feedback was maintained until and during the next trial.

### LFP and difficulty

Critically, difficulty was the least influential parameter, being found in <10% of sites across the considered time windows, and with little to no change in proportion across the window of interest (Fig. 5*d*). Permutation tests revealed only sporadic significance in MCC and mostly for Monkey A. Because RT revealed a significant interaction between difficulty and accuracy (correct or incorrect), we also tested the effect of difficulty on LFP for correct trials only, but this did not change the overall results. Interestingly, LFP varied with RT, and RT differed with difficulty, but we found little LFP variation with difficulty (Fig. 2*b*). In our design, difficulty has only three levels, which might have limited the range of effects. We verified that the LFP modulation with RT remained for each difficulty level: we split trials and tested LFP variations with RT independently for each difficulty level. The effects of RT on LFP remained similar whatever the difficulty level, and were observed for less than 10% of sites. We finally analyzed the effect of difficulty on each tercile of the RT distributions of each difficulty level (i.e., on RT from the first terciles of easy, medium, and hard trials, from the second terciles, etc.), to evaluate whether a difficulty effect could be found only for subgroups of trials with either short or long RT. We observed no such effects, and the analyses revealed only ∼5% of sites with significant LFP modulation by difficulty. In conclusion, this confirmed further the dissociation between RT and difficulty as factors of influence on LFP, the former having significant impact on LFP amplitude but not the later.

We further controlled whether parameters used in the analyses could have influenced these results, in particular the weakness of difficulty effects. Specifically, we checked whether the time-bin size used for analyses (10 ms) influenced the outcome of the analyses and potentially masked effects that would require longer durations to be detectable, especially for the difficulty effect. For instance, one could think that difficulty would be detectable over longer time bins. To verify that the bin size did not influence the outcome of the analyses, we tested the same models (Eq. 4) on data binned in 10, 100, or 500 ms. Longer bins were not tested because of the shorter time between events. The analyses showed that results were qualitatively comparable regardless of the bin size, showing in all cases that the LFP variation was mostly dependent on the factors studied but little with difficulty. We used proportion tests to compare the relative proportions of significant sites for the three factors, to evaluate the relative weight of each factor for each bin size. These revealed that, by increasing bin size, the effect of difficulty decreased, while the effect of touch position increased and the effect of RT remained stable. To evaluated these effects, we tested the relative proportion of sites with significant effect for each factor tested for the three bin sizes. The proportion tests revealed no significant proportion differences between time bins analyses for RT for each area and each monkey, at *p* < 0.05. Regarding the fixed effect of touch position, most (3 of 4) comparisons showed significant proportion differences, at *p* < 0.05, and the data revealed an increasing number of significant sites for larger bin sizes. The difference was nonsignificant (*p* = 0.094) for LPFC in Monkey H. Finally, regarding difficulty, 2 of 4 comparisons revealed significant proportion differences between time bins, at *p* < 0.05, with a decreasing number of significant sites for larger bin sizes (not significant for Monkey H/LPFC, *p* = 0.257, and for Monkey A/LPFC, *p* = 0.050).

We also assessed whether the different tasks and trial factors, including difficulty, modulated specific oscillatory activity, using time-frequency decomposition. We found significant changes in lower frequency (theta and beta bands) associated with touch position and RT, but no consistent significant change for difficulty. Across a total of 8 considered time windows, after cue onset and around lever release (see Materials and Methods), the fraction of sites for which LFP power was modulated by difficulty fluctuated at ∼5%: between 4% and 8.2% for the theta and beta band. In contrast, modulations with RTs were observed in 3.9%-14.3% of sites for the theta band and from 5.3% to 27.0% for the beta band. Finally, a modulation of power with touch position was observed in 4.2%-19% sites for the theta band and from 4.6% to 18.6% for the beta band.

## Discussion

We trained 4 monkeys to a task inducing various levels of behavioral adaptations, on the short (to task difficulty and outcomes) and long terms (to time on task). We found that neural activity in frontal areas correlated little, if any, with difficulty, but strongly reflected time-in-session, RT, touch position, and feedback in the previous and current trial. Thus, the main measures correlating with LFP amplitude were linked to response- and motivational-related factors, corroborating and extending previous research performed on the two frontal areas (e.g., Funahashi et al., 1989; Procyk et al., 2000; Kennerley et al., 2009; Hunt et al., 2018). Here we mainly emphasize the contrast in weights between these factors and difficulty in explaining neural activity variations. The proportion of sites coding for the different variables differed little between MCC and LPFC, apart from feedback valence and touch position.

### Modulation of performance during the categorization task

We defined difficulty *a priori* as the level of accuracy in trials grouped by the nature of the stimulus (angle of cue) used for response. The experimental manipulation of the stimulus to obtain different levels of correct response induced changes in RTs in the 4 monkeys. This is consistent with the literature where, in humans and monkeys, accuracy and RTs (or movement times) covary with difficulty levels (Boudreau et al., 2006; Chen et al., 2008; Pardo-Vazquez et al., 2008; Teichert et al., 2014; Martinez-Garcia et al., 2015; Brechmann and Angenstein, 2019; Habak et al., 2019).

The difficulty-RT correlation differed for correct and incorrect trials, suggesting that changes in RTs might not be solely related to changes in difficulty but also to other factors related to the state of the animal in these different trial types. Specifically, RTs increased with difficulty levels for correct trials, but decreased for incorrect trials. Such effect has been shown previously on movement times (Martinez-Garcia et al., 2015). We assume that for the incorrect easiest trials, the increase of RTs was due mostly to attentional issues and not to a question of increased processing time. The fact that the proportion of incorrect easy trials is small compared with incorrect hard ones goes in that direction.

In addition, 3 of 4 monkeys displayed a slight change in performance after negative versus positive outcomes. This suggests that the outcome in one trial induced sustained changes carried over to the next trial. LFP analyses indeed showed that feedback valence was detectable, for the 2 recorded monkeys, throughout the intertrial interval and even during the next trial. The computation and maintenance of the history of outcomes are considered one important property of the frontal cortex and especially of the MCC (Kennerley et al., 2006; Seo and Lee, 2007; Procyk et al., 2021). Interestingly, in the categorization task, the outcome has no informational relevance for the accuracy in the next trial. And indeed, behavioral data show that a negative outcome tended to be detrimental compared with a positive outcome. This might suggest that also noninformational, emotional, or motivational factors were also carried over and interfered with processing in the next trial.

### Definitions of difficulty

The concept of difficulty is used in many experimental contexts where performance varies with task demand, which could be a problem in itself. The operational definition of difficulty is often weak, and the term has been rather vague in some studies (for discussions, see Paus et al., 1998; Botvinick et al., 2001). A “more difficult task” has been defined as one with a longer latency of response and/or a higher error rate (Paus et al., 1998). In the meta-analysis from Paus et al. (1998), a ranking of tasks by difficulty was equivalent to a ranking of working memory demands. Difficulty can be defined in relation to accuracy, efficiency, performance linked to reaction and response times, and it thus often reflects a consequence on behavior and not a mechanism or a specific property (Li et al., 2018). A high level of difficulty is usually associated with low accuracy and long reaction or response time, suggesting that the cognitive or attentional effort required in more difficult tasks is higher. Thus, in experimental sciences, difficulty is defined by, and must lead to, distinct performance levels. In cognitive neurosciences, the notion of difficulty can also be associated, or confounded, with concepts, such as incongruence, uncertainty, conflict, cognitive effort, and/or task demand. “Task difficulty” is supposed to impact the amount of usable information extracted within a task (Bootsma et al., 2018). “Task conflict” has been defined as any change in task performance induced by distractors (Ebitz and Platt, 2015). Such varied or open definitions make formal interpretations of correlations with neural activity quite hazardous. Finally, “difficulty” can be associated with a potential metacognitive evaluation of the current state of the task at hand (e.g., “I find this difficult”). In this case, difficulty relates to an internal representation of a quality of the current task and not to processes or mechanisms triggered to cope with the current task. In summary, difficulty is not a thing in itself, but a measure of the behavioral expression of task performance. Whenever “difficulty” is used in an experimental design a concrete *a priori* definition based on clear behavioral criteria (e.g., accuracy) should be used to allow comparisons between experiments.

Our study revealed that LFP in MCC and LPFC showed little, if any, relationship with difficulty measured by accuracy levels. The effects found for a minority of recordings in one animal were quite unstable in time. This contrasts with what would be expected given some of the fMRI literature (Shenhav et al., 2014), but is in phase with other data showing strong correlation of frontal BOLD and neural activity with RTs (Nachev, 2011; Kolling et al., 2016). Indeed, RTs correlated with LFP in our experiment. Nevertheless, in monkeys, the effect of difficulty was previously studied with single-unit activity (e.g., in frontal eye field, visual areas V1 and V4, ventral premotor cortex, and locus coeruleus) (Rajkowski et al., 2004; Boudreau et al., 2006; Chen et al., 2008; Pardo-Vazquez et al., 2008; Teichert et al., 2014). Recordings in V1 revealed different unit types with response enhancements and suppressions for different levels of discriminability (and performance) revealing modulations of attentional mechanisms with performance (Chen et al., 2008). Locus coeruleus activity did not vary in amplitude with difficulty (correlated with increased proportion of errors) but in latency, and changes in amplitude were mostly related to RTs (Rajkowski et al., 2004). In frontal eye field, 23% of units were modulated by task difficulty, where difficulty was related to different levels of accuracy in a visual categorization task (Teichert et al., 2014). However, as discussed by the authors, there was no clear and unambiguous interpretation for such a modulation. In summary, “difficulty” can relate to different phenomena, and ill-defined criteria for difficulty might be at the origin of discrepancies in the literature.

### Time on task

Time on task can be defined as a change in vigilance and/or task performance observed during sustained engagement in a task (Boksem et al., 2006; Esterman et al., 2013). Time on task is often associated with changes in RTs, decrement of accuracy, or increase in execution errors (Boksem et al., 2006; Lorist et al., 2009). Neural correlates of time on task have been observed in event-related potentials, BOLD signals, electroencephalography or electrocorticography oscillation power, and single-unit activity (Boksem et al., 2006; Borghini et al., 2014; Stoll et al., 2016b; San-Galli et al., 2018; Müller and Apps, 2019). Single-unit recordings in the ventral mPFC have revealed changes related to motivational and/or control processes (San-Galli et al., 2018). Whether such changes reflect symptoms of fatigue, motivational decrements, or on the contrary compensatory mechanisms remains to be discovered (Sarter et al., 2006; Stoll et al., 2016b). We found that, while RTs increased and accuracy sometimes decreased with time-in-session, LFP in two areas known to contribute to aspects of performance monitoring and cognitive control revealed mostly increased amplitude with time-in-session.

Neural activity changes with time on task can be attributed to several mechanisms that covary during extended task performance: vigilance decrement, increased attentional effort and cognitive control, changes in mood or motivation, satiety in case of food rewarded protocols, etc. Boredom, satiety, and drop in motivation are somewhat difficult to reconcile with increased activity in frontal areas in particular in MCC. Previous analyses of frontal beta oscillation changes (mostly increases) with time-in-session could be better explained by increased compensatory mechanisms, such as increased attentional effort engaged to keep performing the task while fatigue is building (Stoll et al., 2016b). The increased correlations between RT and LFP from the beginning to the end of sessions support this interpretation. However, without dedicated protocols, the basic mechanisms involved are difficult to identify.

### Interpretational issues

Behavioral changes along a session are also reflected in changes in RTs that themselves correlate with changes in frontal neural activity. Changes in neural activity along a session could thus potentially be explained by changes in accuracy and RTs. We included previous feedback and time-in-session in the same models and still found separated effects. We observed that up to 40% of LFP sites presented a significant correlation at some point in trials with time-in-session. LFP amplitudes were not baselined to conserve intertrial changes in signals. However, such an approach seeks a linear change in LFP from beginning to the end of sessions. Yet, previous experiments have shown adaptive reset of time on task effects on frontal neural signals when animals make pauses in work, leading to nonlinear changes within sessions (Stoll et al., 2016b). Other studies have found oscillating performance correlated with changes in frontal oscillation power (Gaillard et al., 2020). Thus, the purely linear descriptor in *glm* might not have been adequate to fully describe these phenomena, and further in-depth analyses are required. Finally, acute microelectrode recordings (as described here) are often impacted by very slow drifts in microelectrode depth because of neural tissue movement relative to the electrode. Such drifts are detectable in single-unit recordings, but their impact on LFP are potential confounds for time on task effects. Yet it is unlikely that such an artifact could explain that the majority of observed changes with time-in-session were positive (i.e., increased amplitude of LFP with time-in-session).

### MCC and LPFC functional differences

MCC and LPFC LFP modulations were very similar in this study. The two main functional differences observed between the two regions seemed to be the response to feedback and the predominance of touch position. MCC recordings revealed an earlier and more frequent response to the outcome than in LPFC (Fig. 7), and showed in proportion more frequent modulations by touch position than in LPFC. This differentiation concurs with previous studies, and supports the predominant role of MCC in feedback processing as well as its functional link with the motor system, the recorded MCC region encompassing the rostral cingulate motor area (Procyk et al., 2016). Yet overall regional differences observed with event related LFPs were not as clear as with single units (Stoll et al., 2016a). This was predictable because of the nature of information one can extract from ERP compared with single units. Moreover, the protocol we used favored the functional coordination of the two regions and did not allow to clearly dissociate them as both contribute to adaptive behavior (Rothe et al., 2011; Stoll et al., 2016a). Previous experiments in monkeys but also fMRI experiments in humans have repeatedly shown coactivation of the two regions in the same conditions (Duncan and Owen, 2000).
